# ERRATUM TO SZCZYPTA ET AL. “DID SAFETY-ENGINEERED DEVICE IMPLEMENTATION CONTRIBUTE TO REDUCING THE RISK OF NEEDLESTICK AND SHARPS INJURIES? RETROSPECTIVE INVESTIGATION OF 20 YEARS OF OBSERVATION IN A SPECIALIST TERTIARY REFERRAL HOSPITAL” (IJOMEH 2024;37(2):234–43)

**DOI:** 10.13075/ijomeh.1896.02554

**Published:** 2024

The original version of the article was published in “International Journal of Occupational Medicine and Environmental Health” 2024; 37(2):234–43, https://doi.org/10.13075/ijomeh.1896.02308.

The authors apologize for the incorrect citation.

## 1. On pages 240–241 the correct references should sound as bolded:

The introduction of legal regulations regarding personnel safety in relation to the provision of services with the use of safe equipment should result in limiting occupational exposure. The first report from the study “Implementation of Council Directive 2010/32/EU in Polish hospitals” was published in 2019 and covered a population of 3954 nurses. The survey-based study revealed that 40% of individuals who experienced workplace injuries or lacerations did not report these incidents [13]. On the other hand, a multicenter study involving 252 Polish hospitals [**26**] demonstrated that every other NSSI was not reported (45.2%). Interestingly, based on data obtained from 26.3% of all Polish hospitals, the authors of the said study estimated the annual average number of NSSIs for nurses, doctors, and paramedics, which should amount to a total of 13 567 cases [**26**]. The authors also calculated rates for the years 2010–2014 for the 252 hospitals. However, the authors do not refer to the implementation of the EU directive, so unfortunately, based on this study, the impact of using SEDs on the occurrence of NSSIs cannot be determined. According to the authors’ best knowledge, the only study evaluating the impact of SED on the occurrence of NSSIs among healthcare workers in Poland is a report from 2019. Despite the results of this study, it should be emphasized that reliable data from all hospitals in Poland are still lacking.

Analyzing the results in comparison to the findings of other authors, both from Poland and other countries, the authors can conclude that the reporting rate of such incidents, although not 100%, is at a fairly satisfactory level. While the number of annual reports varies across different wards, the chosen time frame allowed the authors to observe trends in the phenomenon and confirm that the introduction of safe equipment translates into a lower risk of injuries. Among the available safety-engineered devices, the most commonly used are cannula access devices (82%) and blood collection needles (76%) [13]. In the surveyed hospital, these were: a vacuum blood collection system, safe peripheral venous catheters, as well as safe needles for injections and administering medication. As a result, the strongest downward trend in exposure was observed in conservative wards, with a slight upward trend in surgical wards. The majority of the reported injury cases occurred during surgical procedures, such as suturing and the use of scalpels, for which there is no available equipment with injury pre vention mechanisms. It is worth noting that in the surveyed hospital, which has an average of 110 beds and an average of 203 staff members, it may be easier to implement infection control procedures due to potentially lower anonymity of the staff, resulting in nearly 4 times higher reporting rates compared to the previously cited study by Garus-Pakowska et al. [2]. This confirms the importance of training and organizational factors in implementing effective infection prevention programs, which, combined with the availability of safe equipment, improve workplace safety and hygiene. The significance of training is supported by the findings of de Curli et al. [**27**], who assessed the impact of implementing Directive 2010/32/EU in 97 and 117 Italian hospitals in 2017 and 2021, respectively. In the cited study conducted in 2021, a decrease in the number of training sessions attended by healthcare personnel was observed compared to 2017. This decline was accompanied by a decrease in knowledge levels regarding the prevention of bloodborne infections, as well as a sustained injury rate at a similar level, despite the implementation of multiple safety-engineered devices. Specifically, this involved 89% of SEDs for blood collection and 83% for venous access [**27**]. Training is also necessary when using safe equipment. According to the findings of a survey study by Dulon et al. among 835 healthcare workers, injuries still occur even when SEDs are used. The reasons cited by the respondents include technical problems, unexpected patient movement and problems during disposal [**28**]. The same observations were made by Schurmans et al. [**29**] in a study among 3778 HCWs in a 700-bed hospital in the Netherlands as well as by Grimmond [**30**] in a study involving 7 hospitals in New Zealand. Schurmans et al. [**29**] also did not find a decrease in the percentage of injuries, which they attributed to differences in circumstances . However, a decrease in the number of injuries was confirmed in multicenter studies, similar to the authors’ study [**22, 31**]. Ottino et al. [**31**] analyzed data from 42 acute care hospitals in Piedmont and confirmed an 18% decrease in the number of injuries when using SEDs.

## 2. The correct references − from position 26 − should be as follows:

26. Garus-Pakowska A, Górajski M. Epidemiology of needlestick and sharp injuries among health care workers based on records from 252 hospitals for the period 2010–2014, Poland. BMC Pub Health. 2019;19:634. 10.1186/s12889-019-6996-6.
27. De Carli G, Agresta A, Lecce MG, Marchegiano P, Micheloni G, Sossai D, et al. Prevention from Sharp Injuries in the Hospital Sector: An Italian National Observatory on the Implementation of the Council Directive 2010/32/EU before and during the COVID-19 Pandemic. Int J Environ Res Public Health. 2022;19(17):11144. 10.3390/ijerph191711144.
28. Dulon M, Stranzinger J, Wendeler D, Nienhaus A. Causes of Needlestick and Sharps Injuries When Using Devices with and without Safety Features. Int J Environ Res Public Health. 2020;17(23):8721. 10.3390/ijerph17238721.
29. Schuurmans J, Lutgens SP, Groen L, Schneeberger PM. Do safety engineered devices reduce needlestick injuries? J Hosp Infect. 2018;100(1):99–104. 10.1016/j.jhin.2018.04.026.
30. Grimmond T. UK safety-engineered device use: changes since the 2013 sharps regulations Occup Med. 2019;69(5):352–8. 10.1093/occmed/kqz087.
31. Ottino MC, Argentero A, Argentero PA, Garzaro G, Zotti CM. Needlestick prevention devices: data from hospital surveillance in Piedmont, Italy – comprehensive analysis on needlestick injuries between healthcare workers after the introduction of safety devices. BMJ Open. 2019;9(11):e030576. 10.1136/bmjopen-2019-030576.

